# Spin-orbit coupling enhanced superconductivity in Bi-rich compounds ABi_3_ (A = Sr and Ba)

**DOI:** 10.1038/srep21484

**Published:** 2016-02-19

**Authors:** D. F. Shao, X. Luo, W. J. Lu, L. Hu, X. D. Zhu, W. H. Song, X. B. Zhu, Y. P. Sun

**Affiliations:** 1 Key Laboratory of Materials Physics, Institute of Solid State Physics, Chinese Academy of Sciences, Hefei 230031, People’s Republic of China; 2 High Magnetic Field Laboratory, Chinese Academy of Sciences, Hefei 230031, People’s Republic of China; 3 Collaborative Innovation Center of Microstructures, Nanjing University, Nanjing 210093, China

## Abstract

Recently, Bi-based compounds have attracted attentions because of the strong spin-orbit coupling (SOC). In this work, we figured out the role of SOC in ABi_3_ (A = Sr and Ba) by theoretical investigation of the band structures, phonon properties, and electron-phonon coupling. Without SOC, strong Fermi surface nesting leads to phonon instabilities in ABi_3_. SOC suppresses the nesting and stabilizes the structure. Moreover, without SOC the calculation largely underestimates the superconducting transition temperatures (*T*_*c*_), while with SOC the calculated *T*_*c*_ are very close to those determined by measurements on single crystal samples. The SOC enhanced superconductivity in ABi_3_ is due to not only the SOC induced phonon softening, but also the SOC related increase of electron-phonon coupling matrix elements. ABi_3_ can be potential platforms to construct heterostructure of superconductor/topological insulator to realize topological superconductivity.

Recently, materials with strong spin-orbit coupling (SOC) effect have attracted a great deal of attention due to the resulted novel topological phases. Among those materials, the heaviest group V semimetal Bi-based compounds are mostly investigated^1^. Bi_2_X_3_ (X = Se, Te)^2^,^3^ and ultrathin Bi(111) Films^4^,^5^,^6^ are suggested to be topological insulators. Introducing superconductivity into the topological insulator can make the topological superconductor^7^,^8^. The Majorana fermion is predicted to emerge in topological superconductor, which will deepen our understanding of quantum states of matter in physics and foster innovations in future quantum technologies^7^,^8^,^9^. In principle, the topological superconductivity can show up in doped topological insulators or at the interfaces in a device composed by superconductor and topological insulator^7^,^8^. However, there are only a few systems are reported to be the promising candidates^9^. Doping can introduce superconductivity, making Cu_*x*_Bi_2_Se_3_^10^, Sn_1−*x*_In_*x*_Te^11^, (Pb_0.5_Sn_0.5_)_1−*x*_In_*x*_Te^12^ and Cu_*x*_(PbSe)_5_(Bi_2_Se_3_)_6_^13^ potential platforms to realize topological superconductivity^9^. Very recently a 2D helical topological superconductor was reported to be realized in a heterostructure sample constituting of a Bi_2_Se_3_ film and a s-wave superconductor NbSe_2_^14^. More platforms still need to be explored. Since most reported candidates of topological superconductor are Bi-based compounds^9^, investigating other Bi-based superconductors is necessary.

There is a class of Bi-rich superconductors ABi_3_ (A = Sr and Ba) with simple AuCu_3_ structure (Fig. 1). Polycrystalline ABi_3_ (A = Sr and Ba) and the superconductivity were firstly reported by Matthias and Hulm in 1952^15^. Subsequently, to the best of our knowledge, there were only one experimental report about the polycrystalline samples of Eu doped SrBi_3_ in the following 60 years^16^. First principle calculation without including SOC estimated a superconducting transition temperature (Tc) of 1.8 K for SrBi_3_^17^, which is remarkably smaller than the experimentally measured *T*_*c*_ of ~5.6 K^15^,^16^. Such large deviation was attributed to the disadvantage of the calculation method^17^. Few people have realized that SOC should influence the superconductivity of those compounds in the past years. Very recently, ABi_3_ (A = Sr and Ba) were reinvestigated^18^,^19^. Haldolaarachchige *et al*. prepared the single crystal sample of BaBi_3_ and concluded the physical parameters in detail^18^. Iyo *et al*. investigated superconductivity in polycrystalline sample of Na doped SrBi_3_^19^. However, the role of SOC still has not been discussed.

In this work, we figured out the role of SOC in ABi_3_ (A = Sr and Bi) by theoretical investigation of the band structures, phonon properties, and electron-phonon coupling. We found that without including SOC, strong Fermi surface nesting exists between the electron-pockets at the face centers, which leads to phonon instabilities. SOC suppresses the nesting and stabilize the phonon modes. Moreover, we found the calculation without including SOC largely underestimates *T*_*c*_, while with SOC the calculated *T*_*c*_ are very close to those determined in experiments performed using single crystal samples. Our investigation demonstrates that superconductivity in Bi-rich compounds ABi_3_ (A = Sr and Bi) is strongly enhanced by SOC, which is due to not only the SOC induced softening, but also the SOC related increase of electron-phonon coupling matrix elements. Furthermore, the Bi atoms in the (111) plane of ABi_3_ (A = Sr and Bi) is trigonal, which is very similar to situations in the Bi plane of Bi_2_Se_3_ and ultrathin Bi (111) Films. Therefore, the Bi-rich superconductor ABi_3_ (A = Sr and Bi) can be a potential platform to construct a heterostructure of superconductor/topological insulator to realize topological superconductivity.

## Results

### Theoretical investigation on role of SOC in superconductivity in ABi_3_ (A = Sr and Bi)

The structures of ABi_3_ (A = Sr and Bi) were fully optimized with respect to lattice parameter and atomic positions. For SrBi_3_, the optimized lattice parameter is 5.055 Å, which is in good agreement with experimental value^20^. Nonmagnetic (NM), ferromagnetic (FM), and antiferromagnetic (AFM) states are tested in the system. The magnetic moments of each atom in FM and AFM states are converged to zero, which is consistent with the NM ground state measured in experiment.

**Figure 2:**
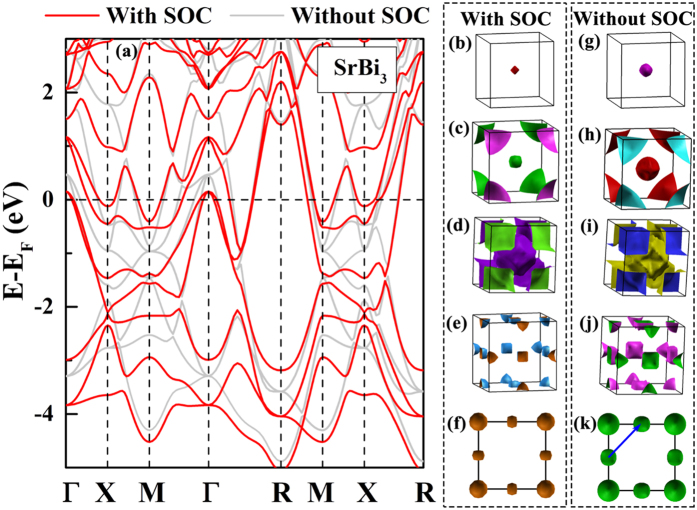
Band structure and Fermi surfaces of SrBi_3_. (**a**) The band dispersion of SrBi_3_ with (the red lines) and without (the grey lines) SOC. (**b**–**e**) are the Fermi surface of SrBi_3_ with SOC, while (**g**–**j**) are those without SOC. (**f**,**k**) are the middle cross sections of (**e**,**j**). The blue arrow in (**k**) denotes the nesting vector *M*.

In Fig. 2(a) we compared the band dispersion of SrBi_3_ with and without including SOC. Because of the high concentration of Bi, one can note that SOC remarkably lifts band degeneracy near Fermi energy (*E*_*F*_) in all the symmetry directions. Four bands cross *E*_*F*_ in each case. SOC shrinks the volumes and marginally changes the shapes of the Fermi surfaces in SrBi_3_, while the locations of the Fermi surfaces are unchanged. More specifically, there are five hole pockets and two electron pockets. Three hole pockets locate around Γ and the rest two hole pockets locate around *R* (Fig. 2(b–d,g–i)). Two electron pockets locate around *M* and *X* points, respectively (Fig. 2(e,j)).

**Figure 3:**
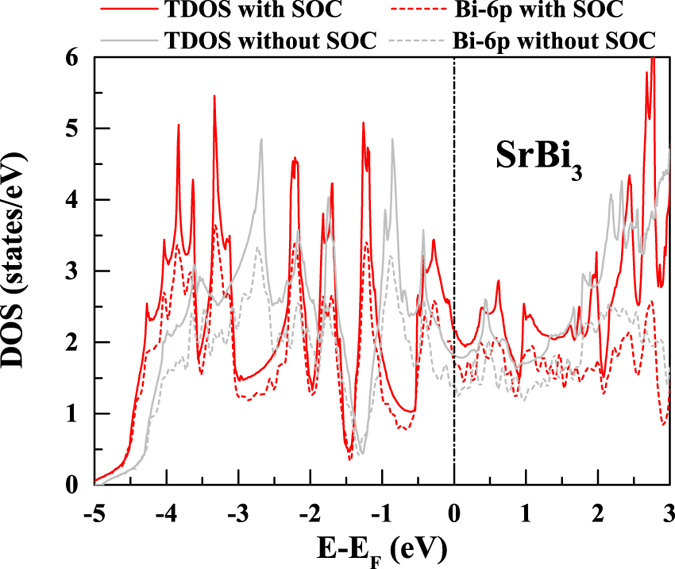
DOS of SrBi_3_. Red color denotes the DOS with and and grey denotes that without SOC. The solid and dashed lines denote the TDOS and the contribution of 6*p* electrons of Bi, respectively.

The density of states (DOS) of SrBi_3_ with and that without SOC were also compared. As shown in Fig. 3, one can note the total DOS (TDOS) near *E*_*F*_ are predominately contributed by Bi-6*p* electrons (Fig. 3). SOC increases the TDOS at *E*_*F*_ (*N*(*E*_*F*_)) by ~20% (Table 1).

**Figure 4:**
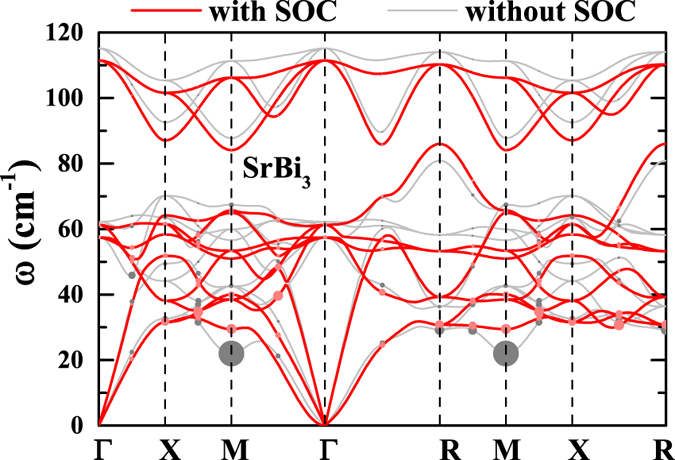
Phonon dispersions of SrBi_3_. The red color denotes the phonon dispersions with SOC while grey denotes that without (grey) SOC. The phonon dispersions are decorated with symbols, proportional to the partial electron-phonon coupling strength 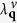.

Figure 4 shows the phonon dispersions of SrBi_3_. In most directions, SOC softens the phonon modes. However, one can note a remarkable softening in the lowest acoustic mode at M point appears when SOC is not included. We attribute such instability to the Fermi surface nesting between the electron pockets around the face centers (*X* point) of the Brillouin zone. As shown in Fig. 2(j,k), when SOC is not included, the electron pockets at face centers in SrBi_3_ show the swelling cubic shape. Large fragments of the pockets at different face centers can be coupled by the nesting vector *M* (Fig. 2(k)). Therefore, stronger instability at M was shown in SrBi_3_ without SOC. On the other hand, SOC changes the shape of such pockets into rectangular hexahedron (Fig. 2(e,f)), which suppresses the nesting and stabilizes the phonon mode at *M*.

**Figure 5:**
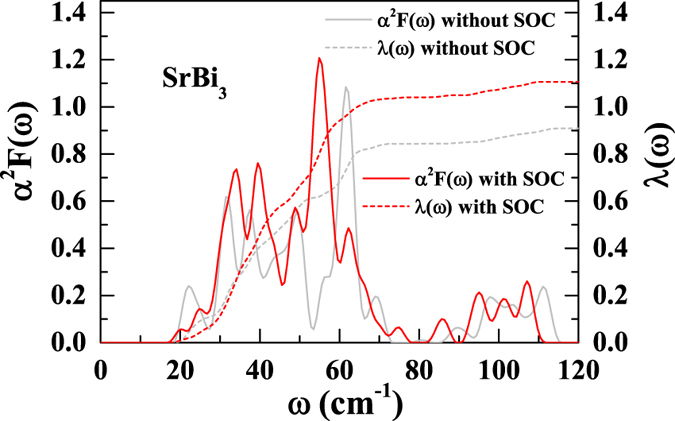
Electron-phonon coupling of SrBi_3_. Eliashberg function (left) and the integrated electron-phonon coupling strength (right) for SrBi_3_ with (red) and without (grey) SOC, respectively.

The electron-phonon coupling can be qualitatively discussed based on Hopfield expression:


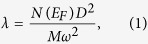


where *D* is the deformation potential, and *M* is the atomic mass. In SrBi_3_, SOC largely increases *N*(*E*_*F*_) and softens most phonon modes. Therefore, one can expect a stronger eletron-phonon coupling when SOC is included. More specifically, Fig. 5 shows the Eliashberg spectral function:


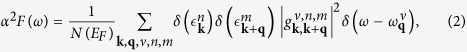


where 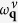 is the phonon frequency, 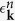 is the electronic energy, and 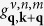 is the electron-phonon coupling matrix element. The total electron-phonon coupling strength is


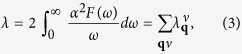


where the electron-phonon coupling strength for each mode 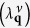 is defined as,


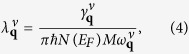


in which 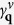 is the phonon linewidth:


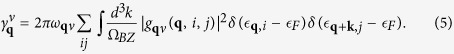



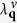
 are visualized as circles in Fig. 4. According to this definition, phonon modes with a lower frequency will lead to stronger electron-phonon coupling. When SOC is not included, the large softening of lowest acoustic mode around *M* point contributes a stronger electron-phonon coupling compared with the case for that SOC is included (Fig. 4). However, it only leads to a small peak between 20 to 25 cm^−1^, which contributes only ~10% of the total electron-phonon coupling strength (Fig. 5). For the modes between 30 to 40 cm^−1^, the *α*^2^*F*(*ω*) peaks with SOC are notably higher than those when SOC is not included, indicating SOC has a sizable enhancement in the electron-phonon coupling matrix elements. Furthermore, since SOC softens the modes in most directions, above 40 cm^−1^ the peaks with SOC become stronger and have lower frequencies. As shown in Fig. 5, SOC largely increased (~20%) the total electron-phonon coupling strength (Table 1).

We estimated *T*_*c*_ based on the Allen-Dynes formula^21^:


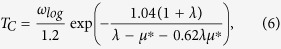


The logarithmically averaged characteristic phonon frequency *ω*_*log*_ is defined as


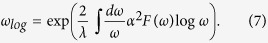


For the Coulomb parameter μ*, we uesd a typical value of 0.10 (a typical value of the Coulomb repulsion between electrons^21^). We listed the calculated *T*_*c*_ and *ω*_*log*_ in Table 1. When SOC is not included, the calculated *T*_*c*_ is only 3.73 K. While, with inclusion of SOC, the calculated *T*_*c*_ is 5.15 K. We also used a derived μ* based on an empirical relation^22^


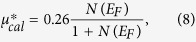


where *N*(*E*_*F*_) is expressed in states/eV/atom. As listed in Table 1, clearly, the choice of μ* does not influence our estimation. Our estimation indicates that the importance of SOC in the superconductivity of SrBi_3_.

**Figure 6:**
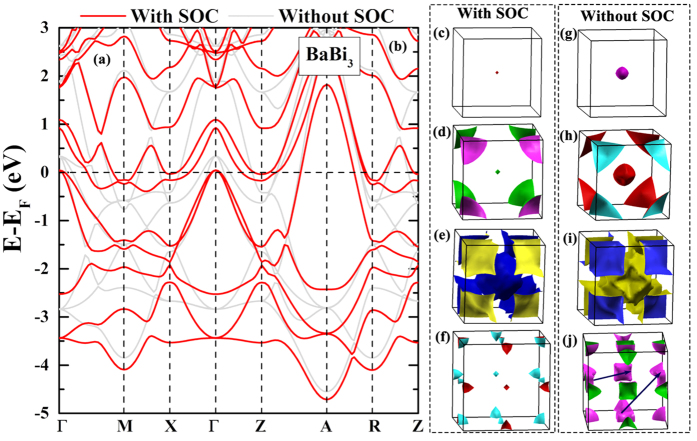
Band structures and Fermi surface of BaBi_3_. (**a**) The band dispersion of BaBi_3_ with (the red lines) and without (grey lines) SOC. (**b**–**e**) are the Fermi surface of BaBi_3_ with SOC, while (**f**–**i**) are those without SOC. The blue arrows in (**i**) denotes the nesting vectors *M* and *R*.

We also calculated the properties of BaBi_3_. The substitution of Sr by Ba changes the crystal from cubic to tetragonal structure. However, the lattice parameters of *a* (5.188 Å) and that of *c* (5.136 Å) are very close to each other. Therefore, the resulted band structure and Fermi surface of BaBi_3_ (Fig. 6) are very similar to those of SrBi_3_. Our calculation is in good agreement with previous report^18^. SOC remarkably lifts the band degeneracy near Fermi energy (*E*_*F*_) in all the symmetry directions of BaBi_3_ as well (Fig. 6(a)). Four bands cross *E*_*F*_, formatting three hole pockets around the body center of the Brillouin Zone (Γ), two hole pockets around the corner of the Brillouin Zone (*A*), and two electron pockets locating at the face centers (*X* and *Z*) and edge centers (*M* and *R*), respectively (Fig. 6(b–i)).

**Figure 7:**
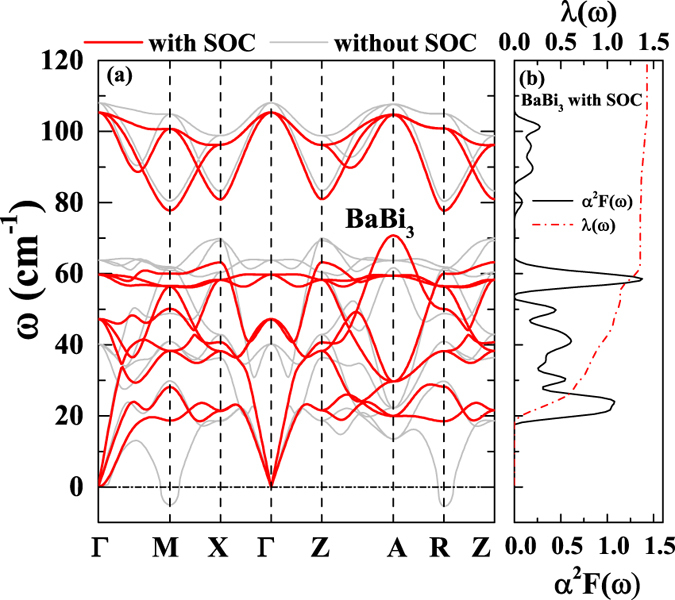
Phonon dispersion and electron-phonon coupling properties of BaBi_3_. (**a**) The phonon dispersions of of BaBi_3_ with (red) and without (grey) SOC. (**b**) Eliashberg function (bottom) and the integrated electron-phonon coupling strength (top) for BaBi_3_ with SOC.

Figure 7(a) shows the phonon dispersion of BaBi_3_. Similar to SrBi_3_, when SOC is not included, the nesting between the electron pockets at different face centers leads to very strong instabilities with imaginary frequency at *M* and *R*. SOC changes such swelling cubic-like electron pockets into spindle-shaped pockets. Therefore, the instabilities are suppressed. In other words, SOC stabilizes the structure of BaBi_3_. The calculated Eliashberg function of BaBi_3_ with SOC is shown in Fig. 7(b). The calculated total electron-phonon coupling strength is 1.43, leading to *T*_*c*_ of 5.29 K (μ* = 0.1) or 5.33 K 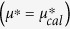. For BaBi_3_ without SOC, since the system is dynamically unstable, we did not estimate its superconductivity.

### Experimental results of single crystal samples

A convenient way to prove our calculation is directly comparing the calculated *T*_*c*_ with the experimentally obtained ones. Although SrBi_3_ has been synthesized sixty years ago, the reported data are mainly based on the SrBi_3_ polycrystalline samples^15^,^16^ and the comprehensive studied on SrBi_3_ single crystal is rarely reported. As we know, the superconductivity is very sensitive to the sample quality of polycrystalline. For example, the reported *T*_*c*_ of MgCNi_3_ in polycrystalline samples varies from 6 K to 9 K^23^. On the other hand, single crystal with good sample quality can reflect the intrinsic properties of the material. The *T*_*c*_ of MgCNi_3_ is proved to be ~6.7 K using single crystal sample, while the physical parameters are measured with higher accuracy in single crystal as well. For the present Bi-rich compounds ABi_3_ (A = Sr and Ba), the studies on single crystal samples are necessary to prove our estimation. Previously Haldolaarachchige *et al*.^18^ prepared the single crystal of BaBi_3_ and measured the physical properties. Our calculated *T*_*c*_ of 5.29 K is very close to the measured *T*_*c*_ of 5.95 K. Here we synthesized the single crystal of SrBi_3_ and performed the related physical measurements.

**Figure 8:**
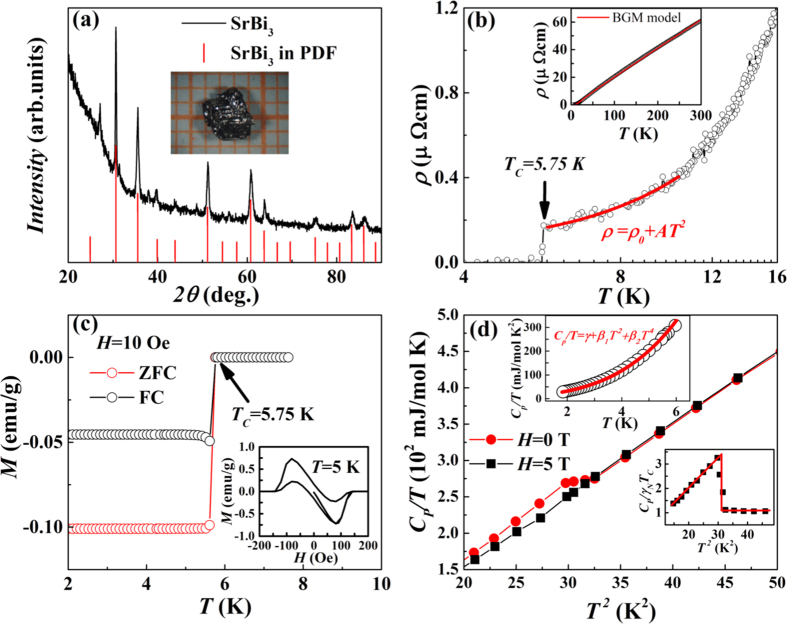
Structure, resistivity, magnetization, and specific heat characterizations of SrBi_3_ single crystal. (**a**) Powder XRD pattern of SrBi_3_ crushed from many single crystals. The red bars are SrBi_3_ in PDF card. The inset shows the studied SrBi_3_ single crystal. (**b**) Temperature dependence of resistivity of the polished SrBi_3_ single crystal. The solid line is the Fermi liquid fitting at the low temperature. The inset shows the Bloch-Grneisen-Mott (BGM) model fitting of the resistivity. (**c**) ZFC and FC magnetic susceptibility of SrBi_3_ single crystal measured at *H* = 10 Oe. The superconducting temperature *T*_*c*_ is 5.75 K. The inset shows the magnetic field dependence of magnetization at *T* = 5 K. (**d**) Heat capacity of SrBi_3_ single crystal measured under *H* = 0 T and *H* = 5 T. The upper inset shows the 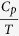 versus *T*, the solid line is fitting according to 
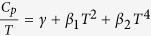
. The lower inset shows the 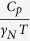 versus *T *^2^.

As shown in Fig. 8(a), single crystals with a size of 3 × 3 × 2 mm^3^ were obtained. Powder XRD measurement indicates the good sample quality. The measured temperature dependences of the resistivity (*ρ*), magnetization (*M*), and specific heat (*C*_*p*_) show the superconducting transition at 5.75 K, which is very close to our estimation. Moreover, the electronic specific heatÎ^3^, which is obtained from the fitting of specific heat based on the relation 
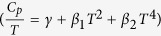
, shows a value of 10.249 mJ/mol K^2^. From the relation 
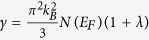
, using the calculated *N*(*E*_*F*_) = 2.17 states/eV, we can estimated the electron-phonon coupling parameter *λ* = 1.005, which is very close to our calculated *λ* = 1.11. The ratio 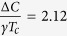 is higher than the BCS weak-coupling limit of 1.43, which also supports our estimated strong coupling scenario. Other fitted physical parameters are presented in the supplementary material. All the measurements verifies our calculation.

## Discussion

In this work, we figured out the role of SOC in ABi_3_ (A = Sr and Bi) by theoretical investigation of the band structures, phonon properties, and electron-phonon coupling. We found that when SOC is not included, strong Fermi surface nesting exists between the electron-pockets at the face centers, which leads to phonon instability. SOC suppresses the nesting and stabilize the phonon modes. Moreover, we found the calculation without including SOC largely underestimates *T*_*c*_. With SOC, the calculated *T*_*c*_ are very close to the *T*_*c*_ determined in measurements on single crystal samples. Our investigation demonstrates that superconductivity in Bi-rich compounds ABi_3_ (A = Sr and Bi) is strongly enhanced by SOC, which is due to not only the SOC induced softening, but also the SOC related increase of electron-phonon coupling matrix elements. Since the arrangement of Bi atoms in the (111) plane of ABi_3_ (A = Sr and Bi) is very similar to that in the Bi plane of Bi_2_Se_3_ and that in ultrathin Bi(111) Films, the Bi-rich superconductor ABi_3_ (A = Sr and Bi) can be a potential platform to construct a heterostructure of superconductor/topological insulator to realize topological superconductivity.

## Methods

The density functional theory (DFT) calculations were carried out using QUANTUM ESPRESSO package^24^ with ultrasoft pseudopotentials. The exchange-correlation interaction was treated with the generalized gradient approximation (GGA) with Perdew-Burke-Ernzerh (PBE) of parametrization^25^. The energy cutoff for the plane-wave basis set was 40 Ry. Brillouin zone sampling is performed on the Monkhorst-Pack (MP) mesh^26^ of 16 × 16 × 16, while a denser 32 × 32 × 32 grid was used in the electron phonon coupling calculations. The Vanderbilt-Marzari Fermi smearing method with a smearing parameter of *σ* = 0.02 Ry was used for the calculations of the total energy and electron charge density. Phonon dispersions were calculated using density functional perturbation theory 27(DFPT) with a 4 × 4 × 4 mesh of *q*-points. To investigate the effect of spin-orbit coupling, fully relativistic calculations were carried out. With the chosen computational parameters, the phonon frequencies are converged within 2 cm^−1^ and *λ* is estimated to be converged to less than 0.01.

Single crystalline specimens of SrBi_3_ were prepared by Bi-self flux. Sr (99.9%, Alfa Aaser) and Bi (99.99%, Alfa Aaser) with mole ratio 1:6 were loaded into alumina crucible, which was placed in quartz tube inside an Ar-filled box. The quartz tubes were sealed under a vacuum. The sealed quartz tubes were slowly heated to 600 °C for 10 hours, then slowly cooling to 330 °C with 3 °C/h. Finally, the excess Bi-flux was removed by decanting. Rectangular shape single crystals with shining surface were observed. The size is about 3 × 3 × 2 mm^3^. The single crystals were kept inside the glove box until characterization. Such handling is necessary to avoid decomposition. Powder X-ray diffraction (XRD) patterns were taken with Cu *K*_*α*1_ radiation (*λ* = 0.15406 nm) using a PANalytical Xpert diffractometer at room temperature. Magnetic, electrical transport and heat capacity measurements were measured using the Quantum Design MPMS-XL5 and PPMS-9. Magnetization measurements under pressure were performed using a pistoncylinder apparatus using the gasket and glycerol as the pressure transmitting medium.

## Additional Information

**How to cite this article**: Shao, D. F. *et al*. Spin-orbit coupling enhanced superconductivity in Bi-rich compounds ABi_3_ (A =Sr and Ba). *Sci. Rep*. **6**, 21484; doi: 10.1038/srep21484 (2016).

## Supplementary Material

Supplementary Information

## Figures and Tables

**Figure 1:**
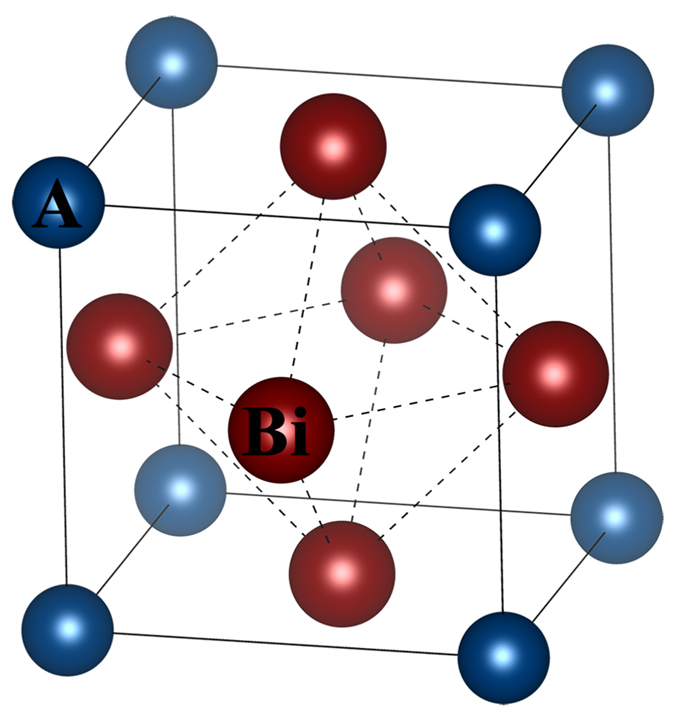
Crystal structure of ABi_3_. Bi-rich superconductors ABi_3_ (A = Sr and Ba) have a simple AuCu_3_ structure, in which A atoms locate at the corners of the unit cell, while Bi atoms locate at the face centers.

**Table 1:** The calculated *N*(*E*_*F*_), *ω*_*log*_, *λ*, derived 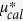 and *T*_*c*_ of ABi_3_ (A = Sr and Bi) with and without SOC.

	*N*(*E*_*F*_) (states/eV)	*ω*_*log*_ (K)	*λ*	*T*_*c*_ (K) (μ* = 0.1)	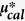	*T*_*c*_ (K) 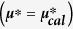
SrBi_3_ without SOC	1.81	63.03	0.91	3.73	0.081	4.14
SrBi_3_ with SOC	2.17	64.04	1.11	5.15	0.091	5.35
BaBi_3_ without SOC	2.02	–	–	–	–	–
BaBi_3_ with SOC	2.40	48.69	1.43	5.29	0.098	5.33

For BaBi_3_ without SOC, since the system is dynamically unstable, *ω*_*log*_, *λ*, and *T*_*c*_ were not calculated.
