# Corrigendum: Spin-orbit coupling enhanced superconductivity in Bi-rich compounds ABi_3_ (A = Sr and Ba)

**DOI:** 10.1038/srep26303

**Published:** 2016-05-31

**Authors:** D. F. Shao, X. Luo, W. J. Lu, L. Hu, X. D. Zhu, W. H. Song, X. B. Zhu, Y. P. Sun

Scientific Reports 6: Article number: 2148410.1038/srep21484; published online: 02192016; updated: 05312016

In this Article, some instances of ‘(A = Sr and Ba)’ are incorrectly written as ‘(A = Sr and Bi)’.

