# Supplementary material for: Spin-orbit coupling enhanced superconductivity in Bi-rich compounds ABi3 (A = Sr and Ba)
Source: Sci Rep. 2016 Feb 19;6:21484. doi: 10.1038/srep21484 (PMC4759591; doi:10.1038/srep21484)
Supplement: Supplementary Information [file srep21484-s1.pdf]

Spin-orbit coupling enhanced superconductivity in Bi-rich compounds ABi_3 (A=Sr and Ba)

D. F. Shao[1,†], X. Luo[1,†], W. J. Lu[1,*], L. Hu[1], X. D. Zhu[2], W. H. Song[1], X. B. Zhu[1], and Y. P. Sun[2,1,3,*]

[1] Key Laboratory of Materials Physics, Institute of Solid State Physics, Chinese Academy of Sciences, Hefei, 230031, China

[2] High Magnetic Field Laboratory, Chinese Academy of Sciences, Hefei, 230031, China

[3] Collaborative Innovation Center of Advanced Microstructures, Nanjing University, Nanjing, 210093, China

[†] The authors contributed equally to this work.

[*] Corresponding author: wjlu@issp.ac.cn and ypsun@issp.ac.cn

Supplemental material

Superconducting parameters of $SrBi_3$

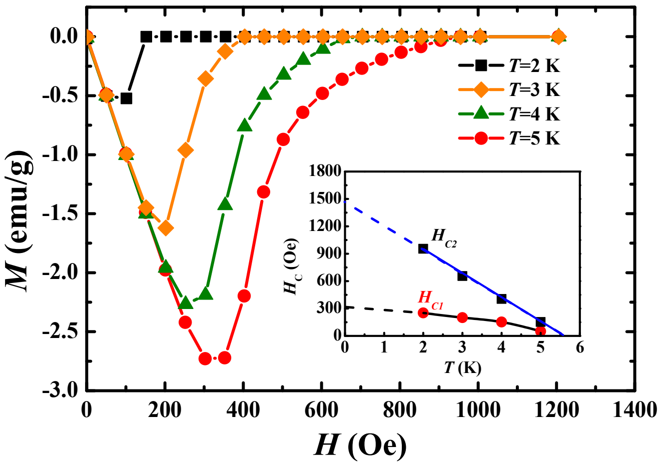

Figure S1: Field dependence of magnetization at different temperatures. Inset: Lower critical field (H_{C1}) and upper critical field (H_{C2}) as a function of temperature. Solid lines are fits to the Eqs. (1) and (2).

Figure S1 shows the magnetic field dependent of magnetization at different temperatures. A typical field dependence of magnetization is observed below T_c. Magnetization linearly increases in magnitude, then decreases after reaching the lower

critical field (H_{C1}), and eventually turns to a paramagnetic state ($M > 0$) above the upper critical field (H_{C2}). The lower critical field H_{C1} can be obtained according to the following equation

$$H_{C1}(T) = H_{C1}(0)[1-(T/T_C)^2]. \qquad (1)$$

A fit to the data in the inset of Fig. 2 (a) yields H_{C1}=280 Oe, which is comparable to that of $BaBi_3$ [1]. The upper critical field H_{C2} is obtained from the magnetization dependent of magnetic field. From the Werthamer-Helfand-Hohenberg (WHH) expression

$$\mu_0 H_{C2} = -0.693 T_C \frac{dH_{C2}}{dT}|_{T=T_c} \ . \qquad (2)$$

The slope is used to calculate $\mu_0 H_{C2} = 0.15\ T$, which is smaller than the weak coupling Pauli paramagnetic limit $\mu_0 H^{Pauli} = 1.82\ T_c = 10.5\ T$. The upper critical field value $\mu_0 H_{C2}$ of $SrBi_3$ single crystal can be used to estimate the Ginzburg-Landau coherence length $\xi_0 = \sqrt{\frac{\Phi_0}{2\pi H_{C2}(0)}} = 460$ Å, where Φ_0 is the magnetic flux quantum. This value is larger than that of other Bi-based superconductors and $MgCNi_3$.[1-3] We can estimate the Ginzburg-Landau parameter $\kappa = \frac{H_{C2}}{\sqrt{2}H_{C1}} \sim$ 1.75. The thermodynamic critical field $H_C(0)=(H_{C1}H_{C2})^{1/2}(ln(k))$~648 Oe. As a result, the magnetic penetration depth $\lambda(0)$ is about 822 Å from $\lambda(0)=\kappa\xi(0)$.

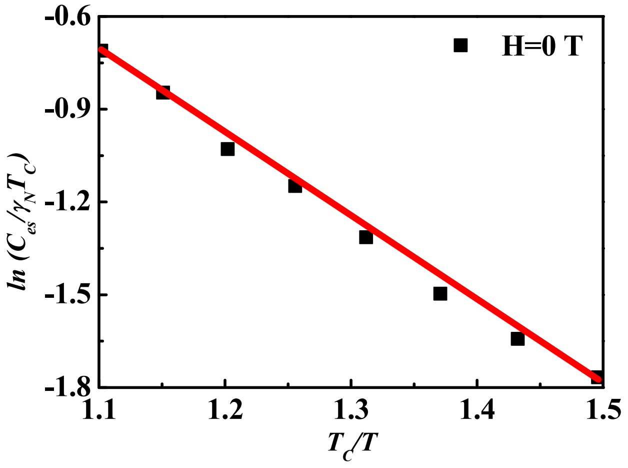

Figure S2: Heat capacity data below T_c. The solid line shows C_e/T calculated by assuming an isotropic *s*-wave BCS gap with $2\Delta/k_B T_c$=5.46.

The exponential behavior of $C_e/T \propto exp(-\Delta/k_BT)$ indicates the energy gap Δ is fully opened. Figure S2 shows the $ln(C_e/\gamma_nT_c)$ versus T_c/T data. The ratio of the gap and the critical temperature is about $2\Delta/k_BT_c$=5.46, which is significantly larger than the BCS value (3.53) in the weak coupling limit, indicating the strong-coupling nature. The analysis suggests that $SrBi_3$ is a BCS-type isotropic-gapped superconductor and *s*-wave paring symmetry dominates the superconducting properties.

Table I: Superconducting parameters of $SrBi_3$ single crystal.

Parameter	Units	$SrBi_3$
T_c	K	5.75
ρ_0	$\mu\Omega\ cm$	0.16
$\mu_0H_{c1}(0)$	Oe	280
$\mu_0H_{c2}(0)$	T	0.15
$\mu_0H_C(0)$	Oe	648
μ_0H^{pauli}	T	10.6
ξ(0)	Å	470
λ(0)	Å	822
κ(0)		1.75
γ(0)	$\frac{mJ}{mol\ K^2}$	7.43
$\frac{A}{\gamma^2}$	$\frac{\mu\Omega\ cm}{(^{mJ}/_{mol\ K})^2}$	5.9×10^{-5}
$\frac{\Delta C}{\gamma T_C}$		2.12
Θ_D	K	111
λ_{ep}		1.02
$\frac{2\Delta(0)}{k_BT_C}$		5.46
$N(E_F)$		1.54

References:

[1] N. Haldolaarachchige, S. K. Kushwaha, Q. Gibson, and R. J. Cava, ***Supercond. Sci. Technol.*** 27, 105001 (2014).

[2] Z. Q. Mao, M. M. Rosario, K. D. Nelson, K. Wu, I. G. Deac, P. Shciffer, Y. Liu, T. He, K. A. Regan, and R. J. Cava, ***Phys. Rev. B***, 67, 094502 (2003).

[3] S. K. Kushwaha, J. W. Krizan, J. Xiong, T. Klimczuk, Q. D. Gibson, T. Liang, N. P. Ong, and R. J. Cava, ***J. Phys.: Condes. Matter***, 26, 212201 (2014).
